# Effects of Pelvic Motion During Robotic-Assisted Gait Training on Balance and Gait Speed in Chronic Stroke: A Randomized Controlled Trial

**DOI:** 10.3390/medicina62050839

**Published:** 2026-04-28

**Authors:** Hyung Joo Lee, Tae Lim Yoon

**Affiliations:** 1 Department of Physical Therapy, Graduate School, Cheongju University, Cheongju 28503, Republic of Korea; taelimyoon@cju.ac.kr; 2 Department of Physical Therapy, Cheongju University, Cheongju 28503, Republic of Korea

**Keywords:** balance, gait, pelvis, rehabilitation, robotics, stroke

## Abstract

*Background and Objectives*: Pelvic fixation during robotic-assisted gait training (RAGT) may limit trunk–pelvis movement and influence functional recovery after stroke. This study investigated whether allowing pelvic motion during RAGT improves balance and gait performance in individuals with chronic stroke. *Materials and Methods*: A single-blind randomized controlled trial was conducted in 49 individuals with chronic stroke (PFG, *n* = 24; PRG, *n* = 25). Participants received Lokomat-assisted gait training (30 min/session, 3 sessions/week for 4 weeks) in addition to conventional therapy. The primary outcome was balance (BBS), and secondary outcomes included DGI, 10 MWT, and pelvic kinematics. Group × time interactions were analyzed using two-way repeated-measures ANOVA. *Results*: Significant group × time interactions were observed for BBS and DGI (*p* < 0.001), indicating greater improvements in the PRG. Gait speed improved significantly over time in both groups (*p* < 0.001), with no significant interaction for the 10 MWT. No significant interaction effects were found for pelvic kinematics, although a group main effect was observed for pelvic tilt. No adverse events were reported. *Conclusions*: Allowing pelvic motion during RAGT was associated with greater improvements in balance and dynamic gait performance compared with pelvic fixation. However, no corresponding changes were observed in pelvic kinematics, suggesting that functional improvements may not be explained by kinematic changes alone.

## 1. Introduction

Gait impairments after stroke are commonly associated with muscle weakness, abnormal tone, and impaired postural control. These deficits often lead to asymmetrical gait patterns, reduced step length, slower gait speed, and altered pelvic motion, which may be associated with reduced walking efficiency and stability [1]. Pelvic rotation and tilt are considered important components of coordinated gait and have been suggested to be associated with trunk–pelvis interaction and mediolateral stability [2]. Impairments in pelvic control may be associated with limitations in functional mobility following stroke.

Conventional rehabilitation approaches, including the Bobath concept, proprioceptive neuromuscular facilitation, and therapist-assisted gait training, typically emphasize trunk and pelvic control during walking [3]. However, these interventions can be therapist-dependent and difficult to standardize over extended treatment periods [4]. Robotic-assisted gait training (RAGT) has been proposed as an alternative approach that provides repetitive and task-specific locomotor practice.

The Lokomat system is widely used in stroke rehabilitation, providing body-weight support and guided lower-limb movements along predefined trajectories [5]. Previous studies have reported improvements in gait speed and balance following Lokomat-based training [6,7]. However, pelvic fixation within the system may restrict natural pelvic motion, particularly rotation and lateral translation [8]. Because pelvic motion has been suggested to be associated with weight transfer and dynamic stability, restricting pelvic motion during training may influence balance-related outcomes [9].

Recent robotic systems incorporating pelvic degrees of freedom have been reported to be associated with improvements in weight shifting during gait [10,11]. Nevertheless, direct comparisons between pelvic fixation and pelvic motion configurations during robotic-assisted gait training, particularly in individuals with chronic stroke, remain limited.

Therefore, this study investigated whether allowing pelvic motion during robotic-assisted gait training is associated with greater improvements in balance performance compared with pelvic fixation in individuals with chronic stroke. Pelvic kinematic variables and gait speed were additionally examined to provide additional context for the observed functional changes. We hypothesized that participants trained with pelvic motion would demonstrate greater improvements in balance-related outcomes, without presuming specific underlying biomechanical mechanisms.

## 2. Materials and Methods

### 2.1. Trial Design

This study was designed as a single-center, assessor-blinded randomized controlled trial conducted at W Convalescent Hospital in Daejeon, Republic of Korea, between July 2025 and October 2025. The study aimed to compare the effects of restricting versus allowing pelvic motion during robotic-assisted gait training (RAGT) using the Lokomat system in individuals with chronic stroke. Pelvic motion in this study was operationally defined as pelvic rotation and pelvic tilt measured during gait.

The study protocol was approved by the Institutional Review Board of Cheongju University (IRB No. 1041107-202504-HR-010-01; approval date: 12 June 2025) and was conducted in accordance with the Declaration of Helsinki. Written informed consent was obtained from all participants prior to enrollment.

The trial was registered in the Korean Clinical Research Information Service (CRIS; registration number: KCT0011572; registration date: 4 February 2026). Registration was completed after study completion; however, the study protocol, eligibility criteria, intervention procedures, outcome measures, and statistical analysis plan were prospectively defined and approved by the Institutional Review Board prior to participant enrollment and were not modified during or after the trial. The protocol submitted to the Institutional Review Board included predefined outcome measures and statistical analysis procedures prior to data collection. Thus, although registration was retrospective, the study was conducted according to a prospectively defined protocol without deviation.

The primary outcome of this study was balance performance assessed using the Berg Balance Scale (BBS). Sample size was calculated a priori using G*Power version 3.1.2 for a two-way repeated-measures ANOVA (group × time interaction). A large effect size (f = 0.40) was assumed based on Cohen’s conventional criteria (1988) and previous systematic reviews of robotic-assisted gait training in stroke populations [12]. With an alpha level of 0.05 (two-tailed), a statistical power of 0.80, and a correlation among repeated measures of 0.50, the required total sample size was estimated to be 40 participants (20 per group). Considering an anticipated dropout rate of 20%, 50 participants were recruited to ensure adequate statistical power.

Participants were randomly assigned to either the Pelvic Fixation Group (PFG) or the Pelvic Release Group (PRG) in a 1:1 ratio. Outcome assessments were performed by a physical therapist blinded to group allocation (assessor-blinded).

### 2.2. Participants

A total of 62 individuals with chronic stroke were assessed for eligibility, of whom 50 met the inclusion criteria and were enrolled in the study. The inclusion criteria were: (a) a confirmed diagnosis of ischemic or hemorrhagic stroke by computed tomography or magnetic resonance imaging, with onset more than three months prior to enrollment; (b) the ability to walk independently or with an assistive device; and (c) a Mini-Mental State Examination (MMSE) score of 23 or higher to ensure the ability to follow instructions [13].

The exclusion criteria were: (a) osteoporosis or fractures of the lower extremities; (b) body weight exceeding 135 kg or height greater than 2 m, exceeding the device limitations; and (c) restricted joint range of motion due to contracture or any medical condition deemed unsuitable for participation by the attending physician.

**Figure 1:**
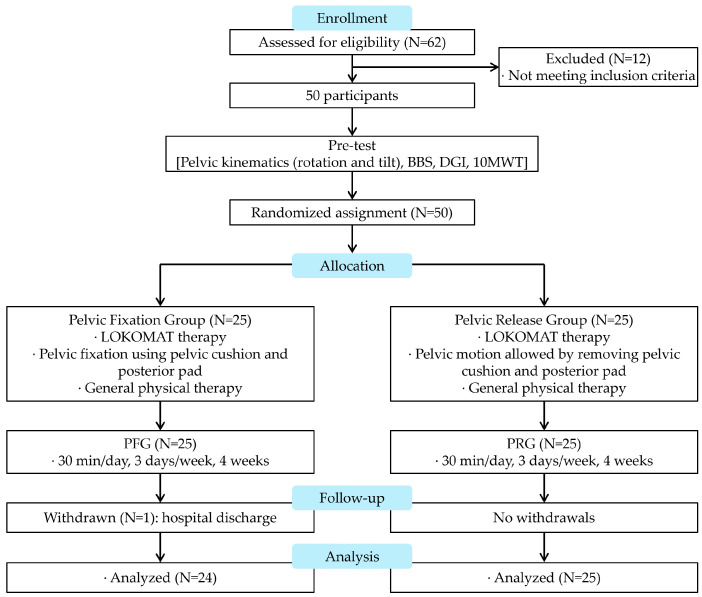
Flow chart.

After screening, the 50 eligible participants were randomly assigned to either the pelvic fixation group (PFG) or the pelvic release group (PRG). One participant in the PFG withdrew due to hospital discharge before completing the intervention and did not provide post-intervention outcome data. No participants in the PRG discontinued the intervention or were lost to follow-up. Consequently, 49 participants (PFG = 24; PRG = 25) completed the intervention and were included in the final per-protocol analysis. as no post-intervention data were available for the withdrawn participant. No participants were excluded after randomization other than the reported withdrawal. The flow of participants through the study is presented in (Figure 1).

### 2.3. Randomization and Blinding

Eligible participants were randomly assigned to either the pelvic fixation group (PFG) or the pelvic release group (PRG) in a 1:1 ratio using a computer-generated simple randomization sequence without stratification. The allocation sequence was generated prior to participant enrollment by an independent researcher who was not involved in participant recruitment, assessment, or intervention delivery.

Allocation concealment was ensured using sequentially numbered, sealed, opaque envelopes. These envelopes were prepared in advance and opened sequentially only after completion of baseline assessments and confirmation of eligibility, thereby preventing foreknowledge of group assignment. The allocation sequence was concealed until group assignment was completed.

Due to the nature of the intervention, which required specific pelvic configuration during robotic-assisted gait training, blinding of participants and treating therapists was not feasible. However, all outcome assessments were conducted by a physical therapist who was blinded to group allocation (assessor-blinded). In addition, statistical analyses were performed by an investigator blinded to group assignment to minimize detection bias.

### 2.4. Intervention

Robotic-assisted gait training was performed using the LokomatPro^®^ system (Hocoma AG, Volketswil, Switzerland). The system consists of a motorized exoskeleton integrated with a treadmill and a dynamic body-weight support (BWS) system. The exoskeleton length and cuff sizes were individually adjusted according to each participant’s anthropometric measurements, and BWS was provided through a harness system.

Participants completed 12 training sessions (30 min per session, three times per week for four weeks). The initial treadmill speed was set at 0.8 m/s, corresponding to the threshold for community ambulation [14], and body-weight support was set at 50% of each participant’s body weight. Guidance force was set at 100% for all participants, allowing the robotic system to fully guide lower-limb movements. This setting was standardized across participants to minimize variability in lower-limb assistance and to focus on the effects of pelvic configuration during robotic-assisted gait training. Guidance force remained unchanged throughout the intervention period. Treadmill speed, body-weight support, and guidance force were kept constant throughout all sessions to ensure standardized training conditions across participants.

**Figure 2:**
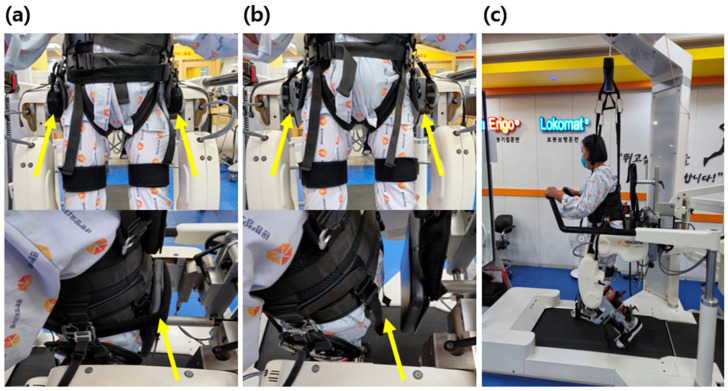
(**a**) Pelvic fixation group (PFG) with restricted pelvic motion using a pelvic cushion and posterior pad; (**b**) pelvic release group (PRG) allowing pelvic motion within the mechanical limits of the Lokomat system following removal of these components; (**c**) robotic-assisted gait training using the Lokomat device (Hocoma AG, Volketswil, Switzerland). The yellow arrows indicate the pelvic cushion and posterior pad. In panel (**a**), the pelvis is stabilized using these components, whereas in panel (**b**), these components are removed, allowing pelvic motion.

The primary difference between groups concerned pelvic configuration. The setup of pelvic fixation and pelvic release conditions is illustrated in (Figure 2). In the pelvic fixation group (PFG), pelvic motion was restricted by applying a pelvic cushion and posterior pad attached to the exoskeleton frame, thereby limiting pelvic motion during gait. In the pelvic release group (PRG), these pads were removed to allow pelvic motion within the predefined mechanical limits of the Lokomat pelvic module. Pelvic configuration was checked and verified prior to each session to ensure consistent application of the intervention conditions throughout the study period.

The mechanical range of pelvic motion was not quantitatively measured during training; therefore, pelvic motion was operationally defined based on device configuration, whereby pelvic motion was either mechanically restricted (PFG) or permitted within the predefined mechanical limits of the Lokomat system (PRG).

Treating therapists supervised all sessions for safety and provided standardized verbal instructions and encouragement; no manual assistance or facilitation of pelvic movement was provided.

For safety, training was discontinued if participants exhibited symptoms meeting the American College of Sports Medicine (ACSM) termination criteria for exercise testing in individuals with stroke. These included severe chest pain, abnormal blood pressure responses (systolic > 260 mmHg or diastolic > 115 mmHg), severe dyspnea, dizziness, syncope, confusion, marked fatigue, or other signs of cardiovascular or neurological instability [15].

In addition to Lokomat training, all participants received 30 min of conventional physical therapy per day, including trunk stabilization exercises, symmetry training, gait practice, weight-bearing training on the hemiplegic limb, and lower-limb strengthening exercises. Post-intervention assessments were conducted after completion of the 12 sessions following a 10 min rest period using the same standardized evaluation procedures.

### 2.5. Outcome Measures

Outcome assessments were conducted at two time points: at baseline (pre-intervention) and immediately after completion of the 12 training sessions (post-intervention). All evaluations were performed by a licensed physical therapist with more than 8 years of clinical experience who was blinded to group allocation.

The primary outcome was balance performance assessed using the Berg Balance Scale (BBS). The BBS is a 14-item clinical scale evaluating static and dynamic balance during functional tasks. Each item is scored on a 5-point scale (0–4), yielding a total score ranging from 0 to 56, with higher scores indicating better balance performance. In stroke populations, the BBS has demonstrated excellent inter-rater and intra-rater reliability (ICC = 0.97–0.98), and scores ≤44 have been associated with increased fall risk [16,17].

Secondary outcomes included dynamic balance, gait speed, and pelvic kinematics. Dynamic balance was assessed using the Dynamic Gait Index (DGI), which consists of 8 items scored from 0 to 3, with a maximum total score of 24 points. Higher scores indicate better dynamic gait stability. The DGI has demonstrated good to excellent reliability in stroke populations (ICC = 0.82–0.92) [18,19].

Gait speed was assessed using the 10-Meter Walk Test (10 MWT). Participants were instructed to walk a total distance of 14 m at a comfortable pace, and the time required to traverse the middle 10 m was recorded, excluding the initial 2 m for acceleration and the final 2 m for deceleration. Three trials were performed, and the average value was used for analysis. The 10 MWT is a reliable and clinically meaningful measure of gait speed in stroke populations, with excellent reliability (ICC > 0.90) and established minimal clinically important differences (MCID) ranging from approximately 0.16 to 0.18 m/s depending on baseline walking ability [14,20].

**Figure 3:**
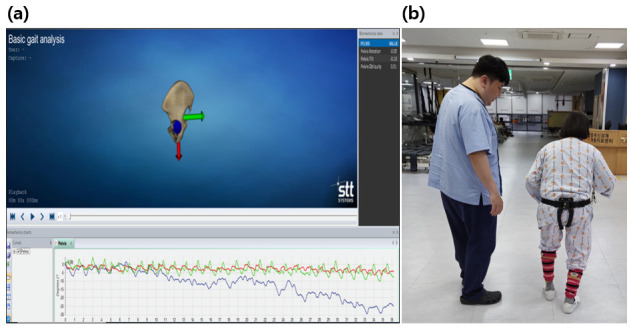
(**a**) iSen gait analysis software displaying pelvic kinematic data; (**b**) gait measurement using a single IMU sensor placed over the sacral region to record pelvic rotation and tilt during walking.

Pelvic kinematics in this study were limited to pelvic rotation and pelvic tilt measured during gait. Pelvic rotation and pelvic tilt were recorded using the iSen (STT Systems, San Sebastián, Spain) inertial measurement unit (IMU) system, which follows the Helen Hayes protocol for gait analysis and standardizes sensor placement and data processing procedures [21]. For pelvic kinematic measurement, a single IMU sensor was secured over the sacral region using a pelvic belt. Participants were instructed to walk naturally over a 10 m walkway while pelvic kinematics were recorded (Figure 3). Pelvic kinematic variables were calculated from data collected during the 10-Meter Walk Test (10 MWT) across the entire walking trial. Positive peak values represented the maximum angular displacement toward one direction (e.g., right rotation or anterior tilt), whereas negative peak values represented the maximum displacement toward the opposite direction (e.g., left rotation or posterior tilt). Accordingly, pelvic rotation and pelvic tilt were expressed as direction-specific peak values (left and right) based on the coordinate system of the IMU. For pelvic tilt, positive values represented anterior tilt and negative values represented posterior tilt. Range of motion was not calculated, as the analysis focused on direction-specific peak values. The iSen IMU system has demonstrated good reliability for lower-limb kinematic assessment during gait (ICC > 0.75) [22]. However, validation studies focusing specifically on pelvic rotation and tilt in stroke populations remain limited.

### 2.6. Statistical Analysis

Statistical analyses were performed using SPSS version 21 (IBM Corp., Armonk, NY, USA). Continuous variables are presented as mean ± standard deviation. The Shapiro–Wilk test was used to assess the normality of baseline data.

Intervention effects were primarily analyzed using a two-way repeated-measures analysis of variance (ANOVA) with time (pre-intervention vs. post-intervention) as the within-subject factor and group (PFG vs. PRG) as the between-subject factor. This model evaluated the main effects of time and group, as well as the group × time interaction. When significant main or interaction effects were observed, simple main effects were examined. Within-group pre–post comparisons were adjusted using the Bonferroni correction.

Although no statistically significant baseline differences were observed, slight imbalances were present in some functional outcomes. Therefore, additional analysis of covariance (ANCOVA) was performed for the Berg Balance Scale (BBS) and Dynamic Gait Index (DGI). In these analyses, post-intervention scores were entered as dependent variables, group was entered as the fixed factor, and baseline scores were entered as covariates to adjust for potential baseline imbalances.

Although the BBS and DGI are ordinal scales, they were analyzed using parametric methods, as these instruments are commonly treated as continuous variables in stroke rehabilitation research when distributional assumptions are reasonably met.

Effect sizes were reported as partial eta squared (η^2^p) for ANOVA results. For within-group changes, standardized mean differences (Cohen’s d) were calculated. Ninety-five percent confidence intervals (95% CI) were reported for mean differences where appropriate to facilitate clinical interpretation.

A total of 50 participants were randomized. One participant withdrew before completing the intervention and did not provide post-intervention outcome data. Therefore, 49 participants (PFG = 24; PRG = 25) completed the study and were included in the final analysis. As no post-intervention data were available for the withdrawn participant, intention-to-treat analysis was not feasible, and a per-protocol approach was applied. No missing outcome data were observed among participants who completed the study, and no data imputation was performed.

The level of statistical significance was set at α = 0.05 (two-tailed).

## 3. Results

### 3.1. Participant Characteristics

A total of 49 participants completed the study (PFG, *n* = 24; PRG, *n* = 25). No statistically significant between-group differences were observed in baseline characteristics, including age, height, weight, body mass index (BMI), MMSE score, onset duration, sex distribution, or paretic side (Table 1).

However, slight differences in some variables (e.g., onset duration) were observed, although these did not reach statistical significance. Overall, the baseline characteristics were generally similar between groups prior to the intervention.

### 3.2. Effects on Balance and Gait Performance

Changes in functional outcomes are presented in (Table 2). Bonferroni-adjusted within-group comparisons indicated that both groups demonstrated significant improvements in BBS and DGI scores, as well as gait speed (10 MWT), after the 4-week intervention (all *p* < 0.001).

Significant group × time interactions were observed for BBS and DGI (both *p* < 0.001), indicating that the magnitude of change differed between groups. Significant overall group effects were also found for BBS and DGI (both *p* < 0.001).

Although no statistically significant baseline differences were observed between groups, slight imbalances were present. Therefore, additional ANCOVA analyses were performed for BBS and DGI with baseline values as covariates. After adjustment, significant group effects remained for both BBS (*p* < 0.001, η^2^p = 0.537) and DGI (*p* < 0.001, η^2^p = 0.377), indicating that post-intervention differences between groups were maintained after controlling for baseline values.

For gait speed (10 MWT), both groups showed significant improvement over time (*p* < 0.001), whereas neither the group × time interaction (*p* = 0.925) nor the overall group effect (*p* = 0.998) reached statistical significance, indicating that gait speed improvements were comparable between groups.

No adverse events or safety-related issues were reported during the intervention period.

### 3.3. Effects on Pelvic Kinematics

Changes in pelvic motion during gait are summarized in Table 3. No significant main effects of time were observed for pelvic rotation left (PRL; *p* = 0.363), pelvic rotation right (PRR; *p* = 0.185), pelvic tilt left (PTL; *p* = 0.665), or pelvic tilt right (PTR; *p* = 0.360).

Similarly, no significant group × time interactions were detected for any pelvic motion variable, indicating that the magnitude of change over time did not differ between the pelvic fixation group (PFG) and the pelvic release group (PRG).

A significant main effect of group was observed only for PTR (*p* = 0.038), indicating a difference between groups irrespective of time. The PRG demonstrated higher PTR values compared with the PFG across both time points. No significant group effects were found for PRL (*p* = 0.257), PRR (*p* = 0.790), or PTL (*p* = 0.227).

## 4. Discussion

This study examined whether allowing pelvic motion during robot-assisted gait training results in different functional outcomes compared with pelvic fixation in individuals with stroke. The main findings were that the pelvic release group (PRG) demonstrated greater improvements in balance and dynamic gait stability than the pelvic fixation group (PFG).

Although gait speed improved significantly within both groups, no significant between-group differences were observed, indicating that the magnitude of improvement was comparable between groups. Importantly, pelvic kinematic variables did not show significant time-dependent changes.

Taken together, these findings suggest that allowing pelvic motion during robot-assisted gait training may be associated with greater improvements in balance-related outcomes, without corresponding changes in pelvic kinematics.

### 4.1. Balance and Dynamic Gait Stability

Both groups demonstrated significant improvements in BBS and DGI scores following the intervention; however, greater improvements were observed in the pelvic release group (PRG) compared with the pelvic fixation group (PFG). These findings are consistent with previous studies reporting that robot-assisted gait training can improve gait symmetry and weight-bearing capacity, thereby contributing to enhanced postural control and balance performance [23,24]. In addition, improved trunk and pelvic control has been associated with reduced asymmetrical loading of the lower limbs [5,25].

Importantly, the magnitude of improvement in BBS should be interpreted in relation to measurement error rather than statistical significance alone. Previous research has reported that the minimal detectable change at the 95% confidence level (MDC95) of the BBS in individuals with chronic stroke ranges from approximately 4 to 6 points, suggesting that changes exceeding this threshold likely represent true change beyond measurement variability [26]. In the present study, the observed improvements in BBS appear to fall within or exceed this range, indicating that the observed changes likely exceed measurement variability and may reflect improvement in postural balance performance.

For the DGI, although a definitive minimal clinically important difference or MDC has not been firmly established in individuals with stroke, prior studies have suggested that changes of approximately 2–3 points may represent detectable or meaningful improvement [18]. Therefore, the observed improvements in DGI may reflect meaningful improvements in dynamic gait performance; however, this interpretation should be made with caution.

Given differences in measurement properties across outcomes, clinical interpretation was based on the most appropriate indices for each measure.

A possible explanation for the greater improvements observed in the PRG is that reduced pelvic constraint may have been associated with less constrained and potentially more natural movement patterns during training. Previous research has shown that robotic gait systems can alter movement patterns due to reduced degrees of freedom and externally imposed constraints [27]. In addition, robotic guidance has been reported to influence muscle activity related to stability and propulsion [28].

Therefore, allowing pelvic motion may provide a less constrained environment that could support more flexible movement strategies during stepping. Although coordination, weight shifting, and trunk–pelvis interaction were not directly measured in the present study, these factors are considered important components of postural control during gait [29]. Accordingly, the present findings may reflect adaptations in movement coordination; however, this interpretation should be considered speculative.

This interpretation is partially supported by previous studies suggesting that restricting pelvic motion during robot-assisted gait training may negatively influence frontal-plane control and gait pattern adaptation [30]. Conversely, robotic systems that allow pelvic degrees of freedom have been associated with more physiological trunk–pelvis coordination during gait-like movements. Taken together, these findings suggest that allowing pelvic motion during robot-assisted gait training may be associated with improvements in balance and dynamic gait stability, potentially through changes in movement coordination rather than increases in pelvic kinematic amplitudes.

### 4.2. Gait Speed

Gait speed increased significantly in both groups, with no significant between-group differences, suggesting a general training effect of robot-assisted gait training (RAGT). Previous studies have similarly reported increases in gait speed following Lokomat training [20,24].

Importantly, the magnitude of change in gait speed should be interpreted in terms of both measurement error and clinical relevance. Previous research has shown that the 10 m walking test demonstrates excellent reliability with low measurement error, with a minimal detectable change at the 95% confidence level (MDC95) of approximately 0.17 m/s in individuals with neurological disorders [20].

In addition, studies in broader populations have suggested that changes of approximately 0.05 m/s represent small but meaningful improvements, whereas increases of approximately 0.10 m/s reflect substantial functional gains in walking ability [31]. In the present study, although gait speed improved in both groups, the extent of change should be interpreted in relation to these thresholds.

The observed improvements in gait speed may be associated with general effects of repeated task-specific training rather than differences in pelvic movement conditions. While several biomechanical factors have been proposed to contribute to increased gait speed, including reduced vertical center-of-mass (COM) displacement [32], increased contribution of pelvic rotation to COM progression [33], increased step length through compensatory mechanisms for limited hip and knee range of motion [5,34], and increased activation of hip extensors and ankle dorsiflexors [28,35], which are often weak in stroke populations [36], these variables were not directly measured in the present study. Therefore, these mechanisms should be considered as possible explanations rather than definitive interpretations.

From a functional perspective, forward propulsion and gait velocity are influenced by multiple factors, including lower-limb force generation and task-specific repetition. Accordingly, improvements in gait speed observed in both groups may reflect general training effects related to repetitive stepping practice and neuromuscular activation, rather than specific effects of pelvic motion.

Taken together, these findings suggest that allowing pelvic motion during RAGT may not directly influence gait speed, but rather that gait speed improvements are primarily driven by general training effects common to both intervention conditions.

### 4.3. Pelvic Motion and Its Functional Relevance

No significant pre–post (time) effects or group × time interactions were observed for pelvic rotation and tilt variables. However, a significant main effect of group was identified only for PTR, favoring the PRG. No significant group effects were observed for PRL, PRR, or PTL. Because the PTR finding was not accompanied by a significant interaction effect, it should be interpreted as a consistent between-group difference rather than a training-induced change in pelvic kinematics.

The absence of significant time-dependent changes in pelvic kinematics, despite improvements in functional outcomes, suggests a dissociation between kinematic variables and functional performance, indicating that functional improvements may not necessarily depend on changes in kinematic amplitudes. Previous studies have indicated that gait control is governed by complex interactions among neural control systems, including automatic spinal locomotor networks, supraspinal motor programs, and multisensory integration, which may allow functional improvements to occur without substantial changes in segmental kinematic amplitudes [29].

The pelvis has been reported to play a role in the intrinsic generation pattern of gait and trunk control via its coupling with lower limbs [30]. For normal gait, a value of roughly 4° of pelvic rotation is necessary for maximum step length, governing velocity and preventing excessive movement of the body’s center of mass that could lead to postural instability [2]. On the other hand, excessive pelvic constraint (as is typically possible in fixed-base robotic devices) can elicit abnormal strategies and limit natural coupling [37].

Importantly, “pelvic release” in this study should be interpreted as allowing pelvic motion within the mechanical limits of the device configuration, rather than unrestricted three-dimensional pelvic motion. Robotic systems that provide guided pelvic DOF may improve trunk–pelvis coupling and mediolateral control without necessarily increasing peak pelvic ROM over a short intervention period [38].

However, coordination, weight shifting, and trunk–pelvis interaction were not directly measured in this study. Therefore, these mechanisms should be considered speculative. In addition, given that multiple kinematic variables were analyzed, the isolated significant finding for PTR should be interpreted with caution.

Therefore, the larger pelvic tilts and overall higher mobility observed in the PRG should be interpreted cautiously, and the superior functional improvement in balance is more plausibly explained by adaptations in coordination and weight-transfer control than by increased kinematic amplitudes per se.

### 4.4. Limitations

This study has several limitations that should be considered when interpreting the findings. First, the relatively small sample size and single-center design may limit the generalizability of the results. Second, the duration of the intervention was relatively short, and no follow-up assessment was conducted; therefore, the long-term effects of the intervention remain unclear.

In addition, trial registration was completed after the study was conducted, which deviates from prospective registration standards and should be considered when interpreting the findings.

Pelvic kinematics were assessed using inertial measurement unit (IMU) sensors. Although IMU-based systems have been reported to demonstrate acceptable validity and repeatability for gait-related measurements, their accuracy in capturing segmental kinematics may be influenced by factors such as sensor placement and algorithm-related processing [39]. In addition, gold-standard motion capture systems were not used in this study. Therefore, the present findings regarding pelvic rotation and tilt should be interpreted with caution.

In addition, multiple kinematic variables were analyzed in this study, and only a single variable (PTR) showed a significant group effect. Thus, the possibility of a type I error cannot be excluded, and this finding should be considered exploratory.

Furthermore, pelvic motion allowance in the PRG reflected a device-defined configuration rather than fully self-initiated pelvic movement. This constraint may have influenced the extent of kinematic adaptation and motor learning during training.

In addition, the use of standardized training parameters (e.g., walking speed, body weight support, and guidance force) may limit ecological validity, as individualized progression is commonly applied in clinical practice.

Finally, coordination, weight shifting, and trunk–pelvis interaction were not directly measured. Therefore, interpretations regarding the underlying mechanisms of functional improvement remain speculative.

A per-protocol analysis was conducted due to missing post-intervention data for one participant, which may introduce potential bias.

Future studies should include larger sample sizes, longer intervention and follow-up periods, and more comprehensive biomechanical assessments, including full three-dimensional kinematics and electromyographic analyses. In addition, further research is needed to clarify the specific contributions of pelvic degrees of freedom to balance control and functional recovery.

## 5. Conclusions

Allowing pelvic motion during robotic-assisted gait training was associated with improvements in balance and dynamic gait performance in individuals with chronic stroke. However, no significant time-dependent changes were observed in pelvic kinematic variables, and gait speed improved similarly in both groups.

These findings suggest that the observed functional improvements may not be directly related to measurable changes in pelvic kinematics. Instead, the improvements may be associated with general training effects, such as task-specific repetition and neuromuscular adaptation. Given the study design and limitations, these findings should be considered preliminary and interpreted with caution.

## Figures and Tables

**Table 1:** General characteristics of the participants at baseline (N = 49).

Item	PFG Mean ± SD (*n* = 24) ^1^	PRG Mean ± SD (*n* = 25)	Test Statistic (t or χ^2^) ^2^	*p* Value
Age (years)	63.42 ± 10.50	62.36 ± 13.41	t = 0.306	0.761
Height (cm)	165.48 ± 8.14	165.50 ± 8.89	t = −0.012	0.991
Weight (kg)	62.41 ± 14.19	62.80 ± 13.43	t = −0.099	0.921
BMI (kg/m^2^)	22.62 ± 3.80	22.79 ± 3.68	t = −0.161	0.873
Onset (months)	20.46 ± 8.70	26.96 ± 13.78	t = −1.965	0.054
MMSE (score)	26.08 ± 2.36	25.76 ± 2.20	t = 0.496	0.622
Sex			χ^2^ = 0.525	0.469
Male	14 (58.3%)	12 (48.0%)		
Female	10 (41.7%)	13 (52.0%)		
Paretic side			χ^2^ = 0.506	0.477
Left	13 (54.2%)	11 (44.0%)		
Right	11 (45.8%)	14 (56.0%)		

^1^ Values are presented as mean ± standard deviation (SD) or *n* (%). ^2^ Independent *t*-tests were used for continuous variables (age, height, weight, BMI, onset duration, and MMSE), and chi-square tests were used for categorical variables (sex and paretic side). BMI, Body Mass Index; MMSE, Mini-Mental State Examination.

**Table 2:** Changes in outcomes between the pelvic fixation and pelvic release groups.

Outcome	Group	Pre (Mean ± SD) ^1^	Post (Mean ± SD) ^1^	Change (Δ) ^2^	Within-Group *p* ^3^	Group × Time *p* (η^2^p) ^4^	Group Effect *p*	Adjusted Group Effect *p* (ANCOVA, η^2^p)
BBS (score)	PFG	29.83 ± 1.71	31.29 ± 1.73	+1.46	<0.001 ***	<0.001 *** (0.881)	<0.001 ***	<0.001 *** (0.537)
	PRG	35.04 ± 0.79	39.96 ± 1.40	+4.92	<0.001 ***			
DGI (score)	PFG	13.33 ± 1.05	14.71 ± 0.95	+1.38	<0.001 ***	<0.001 *** (0.497)	<0.001 ***	<0.001 *** (0.377)
	PRG	16.00 ± 0.82	18.32 ± 0.95	+2.32	<0.001 ***			
10 MWT (m/s)	PFG	0.31 ± 0.13	0.41 ± 0.21	+0.10	<0.001 ***	0.925 (0.000)	0.998	—
	PRG	0.31 ± 0.11	0.41 ± 0.16	+0.10	<0.001 ***			

^1^ Values are presented as mean ± standard deviation (SD). ^2^ Indicates post–pre change. ^3^ Within-group *p* values represent pre–post comparisons adjusted using the Bonferroni correction. ^4^ Effect sizes are reported as partial eta-squared (η^2^p). Significance level: *** *p* < 0.001. BBS, Berg Balance Scale; DGI, Dynamic Gait Index; 10 MWT, 10-Meter Walk Test. ANCOVA was performed for outcomes with baseline differences between groups, using post-intervention values as dependent variables and baseline values as covariates.

**Table 3:** Changes in pelvic motion variables in the pelvic fixation and pelvic release groups.

Pelvic Motion	Group	Pre (Mean ± SD) ^1^	Post (Mean ± SD) ^1^	Change (Δ) ^2^	Time *p* ^3^	Group × Time *p* (η^2^p) ^4^	Group Effect *p*
PRL (°)	PFG	−4.85 ± 3.24	−5.16 ± 3.24	−0.31	0.363	0.614 (0.005)	0.257
	PRG	−5.54 ± 4.37	−6.59 ± 5.28	−1.05			
PRR (°)	PFG	11.46 ± 10.67	7.68 ± 4.38	−3.78	0.185	0.151 (0.043)	0.790
	PRG	9.93 ± 6.49	10.09 ± 6.78	+0.16			
PTL (°)	PFG	−22.19 ± 16.31	−21.30 ± 14.48	+0.89	0.665	0.432 (0.013)	0.227
	PRG	−16.13 ± 13.52	−19.21 ± 13.90	−3.08			
PTR (°)	PFG	17.90 ± 19.44	16.67 ± 15.32	−1.24	0.360	0.623 (0.005)	0.038 *
	PRG	27.57 ± 15.61	23.49 ± 16.75	−4.08			

^1^ Values are presented as mean ± standard deviation (SD). ^2^ Indicates post–pre change. ^3^ Time *p* values represent the main effect of time from repeated-measures ANOVA. ^4^ Effect sizes are reported as partial eta-squared (η^2^p). Significance levels: * *p* < 0.05. PRL, Pelvic Rotation Left; PRR, Pelvic Rotation Right; PTL, Pelvic Tilt Left; PTR, Pelvic Tilt Right. ANCOVA was not performed for pelvic kinematic variables, as no significant baseline differences were observed between the groups.

## Data Availability

The data presented in this study are available from the corresponding author upon reasonable request due to privacy and ethical restrictions.
